# Podocyte specific knock out of selenoproteins does not enhance nephropathy in streptozotocin diabetic C57BL/6 mice

**DOI:** 10.1186/1471-2369-9-7

**Published:** 2008-07-22

**Authors:** Marsha N Blauwkamp, Jingcheng Yu, MaryLee A Schin, Kathleen A Burke, Marla J Berry, Bradley A Carlson, Frank C Brosius, Ronald J Koenig

**Affiliations:** 1 Division of Metabolism, Endocrinology and Diabetes, University of Michigan, Ann Arbor, MI 48109-5678, USA; 2 Cell and Molecular Biology Graduate Program, University of Michigan, Ann Arbor, MI 48109-5619, USA; 3 Division of Nephrology, University of Michigan, Ann Arbor, MI 48109-5676, USA; 4 Department of Cell and Molecular Biology, University of Hawaii at Manoa, Honolulu, HI 96813, USA; 5 Molecular Biology of Selenium Section, Laboratory of Cancer Prevention, Center for Cancer Research, National Cancer Institute, National Institutes of Health, Bethesda, MD 20892, USA

## Abstract

**Background:**

Selenoproteins contain selenocysteine (Sec), commonly considered the 21^st ^genetically encoded amino acid. Many selenoproteins, such as the glutathione peroxidases and thioredoxin reductases, protect cells against oxidative stress by functioning as antioxidants and/or through their roles in the maintenance of intracellular redox balance. Since oxidative stress has been implicated in the pathogenesis of diabetic nephropathy, we hypothesized that selenoproteins protect against this complication of diabetes.

**Methods:**

C57BL/6 mice that have a podocyte-specific inability to incorporate Sec into proteins (denoted in this paper as PodoTrsp^-/-^) and control mice were made diabetic by intraperitoneal injection of streptozotocin, or were injected with vehicle. Blood glucose, body weight, microalbuminuria, glomerular mesangial matrix expansion, and immunohistochemical markers of oxidative stress were assessed.

**Results:**

After 3 and 6 months of diabetes, control and PodoTrsp^-/- ^mice had similar levels of blood glucose. There were no differences in urinary albumin/creatinine ratios. Periodic acid-Schiff staining to examine mesangial matrix expansion also demonstrated no difference between control and PodoTrsp^-/- ^mice after 6 months of diabetes, and there were no differences in immunohistochemical stainings for nitrotyrosine or NAD(P)H dehydrogenase, quinone 1.

**Conclusion:**

Loss of podocyte selenoproteins in streptozotocin diabetic C57BL/6 mice does not lead to increased oxidative stress as assessed by nitrotyrosine and NAD(P)H dehydrogenase, quinone 1 immunostaining, nor does it lead to worsening nephropathy.

## Background

Selenium, a trace element, is found in the amino acid selenocysteine (Sec) and is cotranslationally incorporated into the protein polypeptide chain via the codon UGA. Although UGA generally signals translation termination, mRNAs that contain a conserved SECIS element in their 3' untranslated regions are able to decode UGA as Sec [1]. Sec is synthesized on a unique tRNA [2,3], termed tRNA^[Ser]Sec ^because it is first aminoacylated by serine, which is then converted to selenocysteine. Since this is the only tRNA that supports Sec incorporation into proteins, the absence of tRNA^[Ser]Sec ^results in protein chain termination instead of Sec incorporation. As discussed below, selenoproteins are enzymes with Sec in the active site. Therefore, even if a truncated protein lacking Sec is stable, it will not be biologically active. Thus, the absence of tRNA^[Ser]Sec ^results in complete functional selenoprotein deficiency. Whole mouse homozygous deletion of the tRNA^[Ser]Sec ^gene *Trsp *is embryonic lethal [4]. However, the generation of mice carrying *Trsp *alleles flanked by *lox*P sites has allowed the study of organ-specific deletion of selenoprotein synthesis [5].

The human genome encodes 25 selenoproteins and the mouse genome 24 [6]. Many selenoproteins function as antioxidant enzymes or in redox signaling. Examples of selenoproteins with these activities include the glutathione peroxidases (Gpx) and thioredoxin reductases (Trxr) (reviewed in [7]). Other selenoproteins such as selenophosphate synthetase and selenoprotein P indirectly support those activities by functioning in Sec synthesis and selenium transport and storage [7].

Superoxide is a highly reactive and potentially toxic oxidant produced during mitochondrial respiration and by several cytoplasmic enzymes such as NAD(P)H oxidase. In diabetes, hyperglycemia induces overproduction of superoxide via the mitochondrial electron transport chain as well as by increased NAD(P)H oxidase activity [8] leading to oxidative stress [9]. Oxidative stress is thought to play an important role in the progression of diabetic complications, including nephropathy [10]. Podocyte (glomerular epithelial cell) damage is central to the development of diabetic nephropathy [11]. An increase in glomerular oxidative stress occurs early in diabetic nephropathy and enhanced mitochondrial and cytoplasmic oxidant stress leads directly to apoptosis in podocytes exposed to high extracellular glucose [12]. It has been shown that injury to diabetic kidneys is reduced in transgenic mice that over express superoxide dismutase (SOD1) [13]. In addition, streptozotocin (STZ) diabetic mice developed increased oxidative stress and kidney damage when subjected to a selenium deficient diet [14]. However, a recent study of diabetic Gpx1 homozygous null mice found that Gpx1 was not protective against renal injury [15].

C57BL/6 mice are relatively resistant to the development of diabetic nephropathy [16]. Given the evidence supporting a role of oxidative stress in diabetic nephropathy and the role of selenoproteins in protecting against oxidative stress, we postulated that podocyte selenoproteins protect against the development of diabetic nephropathy in C57BL/6 mice. To test this hypothesis, we created a podocyte specific knock out of all selenoproteins (PodoTrsp^-/-^) in C57BL/6 mice, induced diabetes with STZ, and examined the mice for progression of diabetic nephropathy. Contrary to our hypothesis, we found the PodoTrsp^-/- ^mice did not develop increased nephropathy.

## Methods

### Targeted Inactivation of the Selenocysteine tRNA^[Ser]Sec ^gene *Trsp *in Podocytes

C57BL/6 transgenic mice expressing Cre recombinase driven by the 2.5 kb human podocin (*NPHS2*) promoter were obtained from L.B. Holzman (University of Michigan, Ann Arbor, MI) [17]. The expression of Cre recombinase in these mice does not cause glomerular abnormalities [17-19]. Podocin-Cre mice were mated with C57BL/6 mice in which both *Trsp *alleles are flanked by *lox*P sites, denoted *Trsp*^L/L ^[5]. The resulting podocin-Cre;*Trsp*^L/+ ^mice were mated with *Trsp*^L/L ^mice to generate podocin-Cre;*Trsp*^L/L ^mice, denoted in this paper as PodoTrsp^-/-^. Littermates of the genotype *Trsp*^L/L ^were used as controls. Genotyping was performed by PCR using the following oligonucleotide primers for *Trsp*: 5'-CAA AAC CTC GCC TCC AAG TGA C-3' and 5'-TGT GAG ACG ACC TTC TAT GCT CG-3'; and for Cre: 5'-GCG GTC TGG CAG TAA AAA CTA TC-3' and 5'-GTG AAA CAG CAT TGC TGT CAC TT-3'. The PCR program used for *Trsp *detection is as follows: step 1, 95°C for 5 min; step 2, 95°C for 15 sec; step 3, 64°C for 30 sec; step 4, 68°C for 2 min; step 5, 30 repetitions of steps 2 to 4. The PCR program used for Cre detection is as follows: step 1, 95°C for 5 min; step 2, 94°C for 30 sec; step 3, 51°C for 45 sec; step 4, 72°C for 1 min; step 5, 35 repetitions of steps 2 to 4. All animal care and handling procedures were approved by the University of Michigan Committee on Use and Care of Animals.

### Induction of diabetes

Ten week old male PodoTrsp^-/- ^and *Trsp*^L/L ^(as controls) mice were fasted for 4 hours and then injected intraperitoneally with 50 mg/kg STZ (Sigma) or vehicle for 5 consecutive days according to the low dose protocol of the Animal Models of Diabetic Complications Consortium . STZ was prepared in freshly made 100 mM sodium citrate buffer pH 4.5 at 7.5 mg/ml and used within 15 minutes. For each experiment, the number of mice used is stated in the figure legend.

### Immunohistology

Immunoperoxidase staining for Wilms' tumor homolog (Wt1) and Gpx1 was performed on 2% paraformaldehyde-lysine-periodate (PLP) fixed kidney cryostat consecutive sections (3 μm). PLP-fixed tissue sections were incubated for 2 hrs at 90°C in Retrieve-All 1 (Covance), followed by 3% hydrogen peroxide in methanol for 30 mins and 1.5% normal rabbit serum in 3% BSA, 0.1% Tween 20 in 1× PBS for 20 mins according to Vectastain Elite ABC kits-Goat IgG and Sheep IgG. Sections were then incubated with primary antibodies at 1 μg/μl of either goat anti Wt1 polyclonal (1:200) (Santa Cruz) or sheep anti Gpx1 polyclonal (1:4,000) (Novus Biologicals) for 1 hr at room temperature. After washes in PBS, sections were incubated in biotinylated secondary antibodies (biotinylated anti sheep IgG or biotinylated anti goat IgG) for 1 hr at room temperature, followed by incubation with ABC reagent for 1 hr at room temperature (Vectastain Elite ABC kits-Sheep IgG and Goat IgG). Sections incubated with anti Wt1 polyclonal antibody were developed using Vector SG (Vector) for 5 mins to produce a gray color and sections incubated with anti Gpx1 polyclonal antibody were developed using Vector NovaRED (Vector) for 5 mins to produce a red color.

To assess oxidative stress, immunoperoxidase staining was performed on PLP fixed, paraffin kidney sections. Consecutive 3 μm sections were stained with rabbit anti nitrotyrosine (1:100) (Millipore) or goat anti Wt1 (1:300). As a negative control, the anti nitrotyrosine antibody was preincubated with 25 μM nitrotyrosine for 1 hour and 25 μM nitrotyrosine also was included during the primary antibody incubation. To assess whether antioxidant enzymes might be induced to compensate for the loss of selenoproteins, consecutive 3 μm sections were stained for Wt1 or NAD(P)H dehydrogenase, quinone 1 (NQO1) using rabbit anti NQO1 (1:100) (Abcam). The general procedures for these immunostainings were as described above, except that the sections stained for nitrotyrosine and NQO1 were developed with 3,3'-diaminobenzidine. All immunostainings for nitrotyrosine, as well as all for NQO1 and Wt1, were done at the same time and were photographed under identical conditions. Adobe Photoshop was used to adjust the color of the Wt1 images to facilitate identification of podocytes when the images were merged.

### Biochemical Analysis

Glucose levels in fresh blood obtained between 9 and 10 AM from the tail veins of nonfasted mice were measured once a month by glucometer (Therasense). Urine was collected at 3 and 6 months of diabetes from mice housed in metabolic cages for 4 hrs. Elisa kits-Albuwell M and Creatinine Companion (Exocell) were used to measure urine levels of albumin and creatinine.

### Histology and Morphometry

PLP-fixed tissue paraffin sections (3 μm) were stained with periodic acid-Schiff's (PAS) reagent to identify kidney structure and hematoxylin to visualize cell nuclei. Microscopic images of glomeruli (10 images per sample) were taken at 20× magnification and used for calculating the mesangial matrix cell volume per glomerular tuft. The mean area of each glomerular tuft was measured by manually tracing the glomerular outline on a computer screen and calculating that area by computerized morphometry using Metamorph 6.1 (Molecular Devices) at a final magnification of 150×. The mesangial matrix cell volume was then quantified by measuring the area of the glomerulus stained pink to purplish red (PAS positive) using Metamorph 6.1. Percent of mesangial matrix was calculated as (area PAS positive/total glomerular tuft area) multiplied by 100.

## Results

### Deletion of selenoproteins in PodoTrsp^-/- ^podocytes

**Figure 1:**
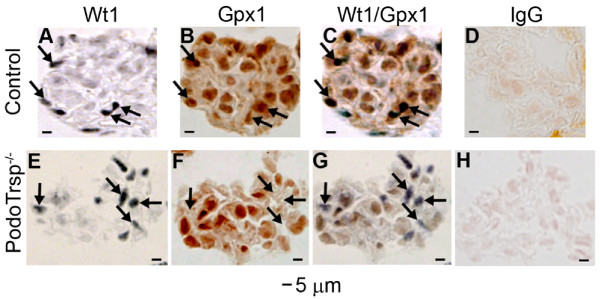
**Immunoperoxidase staining demonstrating a reduction in podocyte Gpx1 expression in PodoTrsp^-/- ^mice**. Consecutive 3 μm kidney sections were stained from control (*Trsp*^L/L^) mice (A-D) and PodoTrsp^-/- ^mice (E-H). A, E Anti-Wt1 antibody staining to identify podocytes (arrows). B, F Anti-Gpx1 antibody staining (arrows indicate the same cells as in A, E). C, G Merge of Wt1 and Gpx1 sections from A, B and E, F, respectively. C, Arrows indicate podocytes from control mice express both Wt1 and Gpx1. G, Arrows indicate podocytes from PodoTrsp^-/- ^mice lose Gpx1 expression. D, H stained with normal sheep IgG as a negative control for the Gpx1 antibody.

To demonstrate the knockout of podocyte selenoproteins, consecutive kidney sections from control and PodoTrsp^-/- ^mice were immunostained for Wt1 to identify podocytes (Fig. 1A &1E) and Gpx1 to identify a selenoprotein (Fig. 1B &1F). Gpx1 is ideal for this purpose because, in the absence of Sec incorporation, Gpx1 mRNA is degraded by nonsense mediated decay, so that very little truncated Gpx1 protein (lacking Sec) is made [20,21]. A merge of the Wt1 and Gpx1 immunostainings shows that control mice express Gpx1 in podocytes (Fig. 1C), whereas expression is very low to nil in PodoTrsp^-/- ^podocytes (Fig. 1G).

### PodoTrsp^-/- ^and control mice developed similar degrees of hyperglycemia and body weight changes in response to STZ

**Figure 2:**
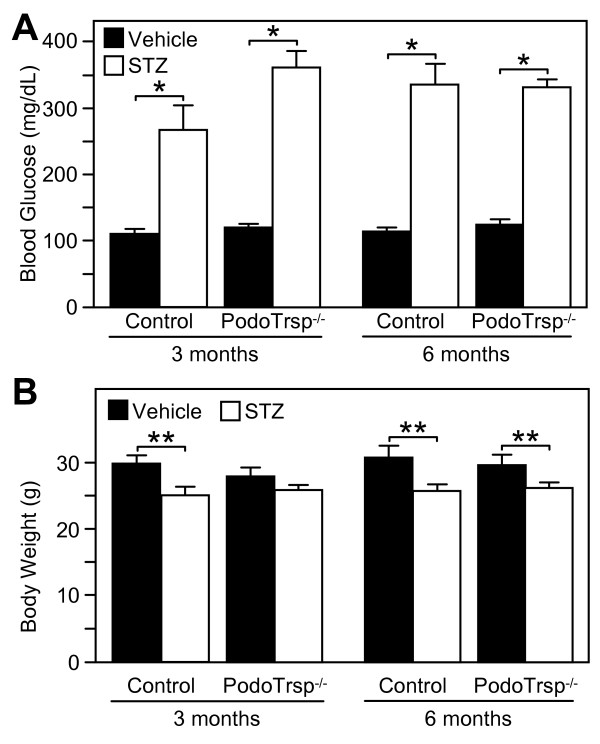
**Blood glucose and body weight in PodoTrsp^-/- ^and control (*Trsp*^L/L^) mice**. A, blood glucose levels and B, body weight 3 and 6 months after the onset of STZ-induced diabetes (or after vehicle injection). Control mice, vehicle n = 10, STZ n = 7; PodoTrsp^-/- ^mice, vehicle n = 13, STZ n = 12. *P *values were determined by two-tailed *t *test. *P < 0.01, **P < 0.001.

STZ diabetes was induced in male PodoTrsp^-/- ^and control mice. The blood glucose levels for STZ treated PodoTrsp^-/- ^and control mice were similar at 3 and 6 months of diabetes (Fig 2A). STZ treated mice also showed a decrease (means ± SEM; P < 0.01) in body weight compared to vehicle treated mice at 3 and 6 months of diabetes (Fig 2B).

### PodoTrsp^-/- ^mice do not have an increased susceptibility to nephropathy after 6 months of diabetes

**Figure 3:**
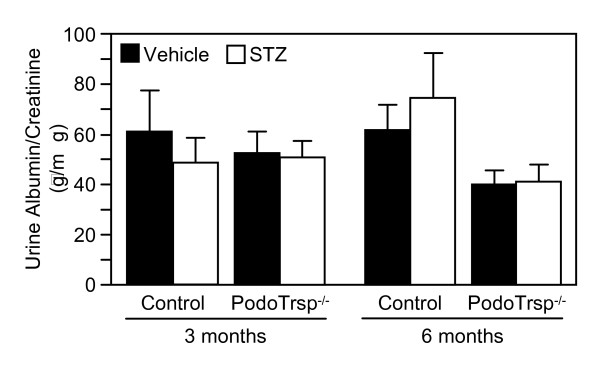
**Urinary albumin/creatinine ratios are not increased in PodoTrsp^-/- ^versus control (*Trsp*^L/L^) mice**. Three months after the onset of diabetes (or after vehicle injection): control mice, vehicle n = 10, STZ n = 7; PodoTrsp^-/- ^mice, vehicle n = 12, STZ n = 13. Six months after the onset of diabetes (or after vehicle injection): control mice, vehicle n = 8, STZ n = 6; PodoTrsp^-/- ^mice, vehicle n = 9, STZ n = 13.

**Figure 4:**
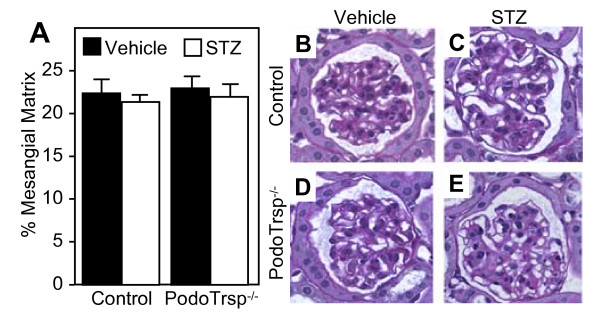
**PodoTrsp^-/- ^and control (*Trsp*^L/L^) mice have similar mesangial matrix volumes**. A, Percent of mesangial matrix in glomeruli from control and PodoTrsp^-/- ^mice after 6 months of diabetes (or after vehicle injection). Control mice, vehicle n = 6, STZ n = 6; PodoTrsp^-/- ^mice, vehicle n = 6, STZ n = 6. B-E, PAS staining of glomeruli.

Microalbuminuria was assessed to evaluate diabetic renal damage. The albumin to creatinine ratios were not increased in PodoTrsp^-/- ^versus control mice after 3 or 6 months of diabetes, and in fact were similar to the levels seen in non-diabetic mice (Fig 3). To further explore diabetic nephropathy, mesangial matrix expansion was analyzed. PAS and hematoxylin staining of kidney sections again showed no significant differences between PodoTrsp^-/- ^and control mice after 6 months of diabetes (Fig 4).

### PodoTrsp^-/- ^mice do not have increased glomerular or podocyte oxidative stress after 6 months of diabetes

**Figure 5:**
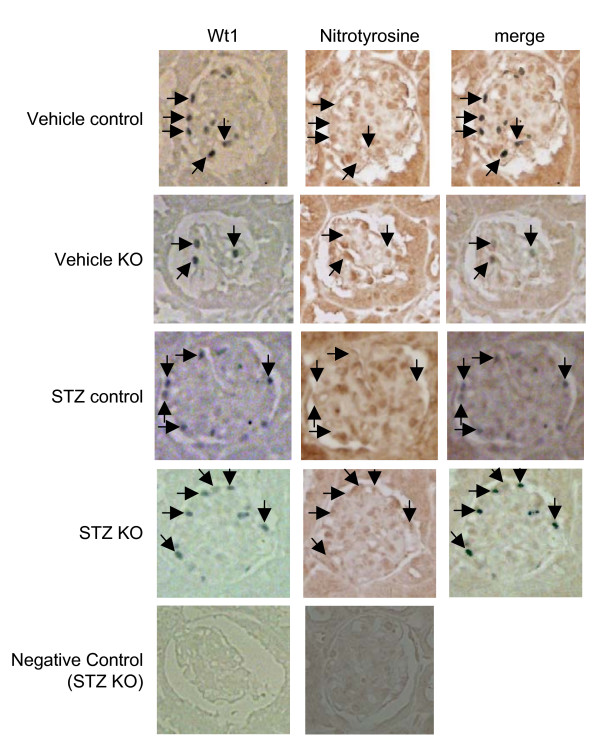
**Immunoperoxidase staining demonstrating similar levels of nitrotyrosine in podocytes of PodoTrsp^-/- ^and control (*Trsp*^L/L^) mice**. Consecutive 3 μm kidney sections from vehicle control, vehicle KO (PodoTrsp^-/-^), STZ control, and STZ KO (PodoTrsp^-/-^) mice were stained for nitrotyrosine or Wt1 (to identify podocytes). Arrows indicate selected podocytes in the Wt1 and merged images, and indicate the identical positions in the nitrotyrosine images. Negative control staining for Wt1 utilized normal IgG in place of the primary antibody, and for nitrotyrosine utilized coincubation of the primary antibody with nitrotyrosine.

**Figure 6:**
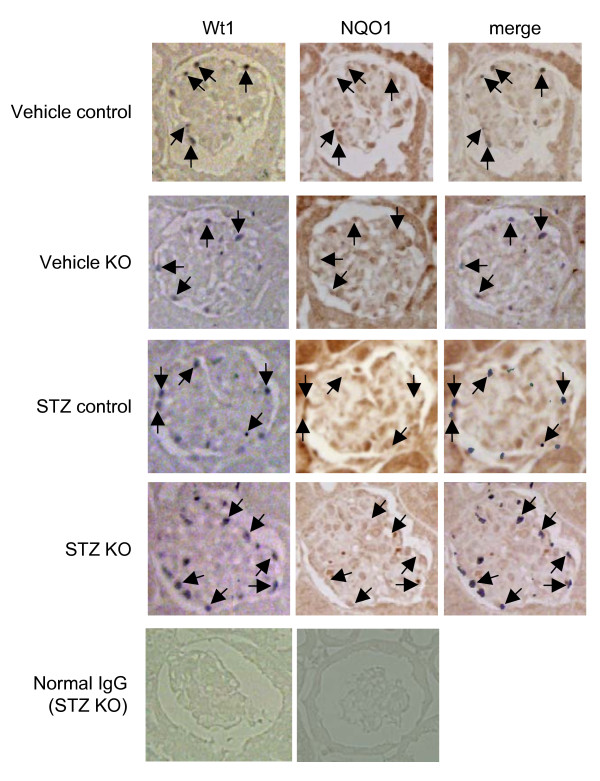
**Immunoperoxidase staining demonstrating similar levels of NQO1 in podocytes of PodoTrsp^-/- ^and control (*Trsp*^L/L^) mice**. Consecutive 3 μm kidney sections from vehicle control, vehicle KO (PodoTrsp^-/-^), STZ control, and STZ KO (PodoTrsp^-/-^) mice were stained for NQO1 or Wt1 (to identify podocytes). Arrows indicate selected podocytes in the Wt1 and merged images, and indicate the identical positions in the NQO1 images. Incubations with normal IgG in place of the primary antibodies served as negative controls.

Consecutive 3 μm kidney sections from control and PodoTrsp^-/- ^mice were immunostained for nitrotyrosine as a marker for oxidative stress and for Wt1 to identify podocytes. No differences were observed between the podocytes of PodoTrsp^-/- ^and control mice for nitrotyrosine immunostaining (Figure 5). Consecutive 3 μm kidney sections also were stained for Wt1 and NQO1 as an antioxidant enzyme that could be induced to compensate for the loss of selenoproteins (Fig 6). Again, no differences were observed between the podocytes of PodoTrsp^-/- ^and control mice for NQO1 immunostaining.

## Discussion

Although multiple lines of evidence suggest an important role for oxidative stress in the pathogenesis of diabetic complications including nephropathy [9-14], the precise role of oxidative stress is not known. In addition, there are many endogenous factors that potentially can counteract oxidative stress, and it is not known which of these are most important in protecting against diabetic complications.

In the diabetic kidney, oxidative stress coincides with podocyte apoptosis [12]; hence, podocyte antioxidant proteins are expected to be important in protecting against diabetic nephropathy. Given the toxicity of uncontrolled oxidative stress, it is likely that substantial redundancy exists in the functions of antioxidant proteins. The majority of selenoproteins with known functions are involved in the management of oxidative stress. This includes four selenoprotein glutathione peroxidases in rodents and three thioredoxin reductases, and likely includes numerous others such as selenoproteins H, K, P, R, S and W ([22] and reviewed in [7]). Thus, we hypothesized that selenoproteins as a group protect against diabetic nephropathy, and that deletion of all selenoproteins would reveal their importance by preventing compensatory effects. We tested this hypothesis by deleting the tRNA^[Ser]Sec ^gene *Trsp *in podocytes of C57BL/6 mice. The choice of mouse strain was dictated by the fact that both the podocin-Cre and *Trsp*^L/L ^genotypes were on pure C57BL/6 backgrounds. The fact that diabetic C57BL/6 mice are relatively resistant to nephropathy [16] also made this a logical choice, allowing us to test whether selenoproteins at least in part underlie this resistant phenotype.

The loss of podocyte selenoproteins did not result in increased nitrotyrosine staining as a marker for oxidative stress, nor did it enhance the development of diabetic nephropathy as assessed by microalbuminuria and mesangial matrix expansion. Our findings extend those of de Haan et al [15], who showed that deletion of Gpx1 alone did not enhance diabetic nephropathy.

The data suggest that podocyte selenoproteins are not important in the protection against diabetic nephropathy, or that remaining antioxidant mechanisms can compensate. However, we did not find increased expression of the antioxidant enzyme NQO1. Our results may not extend to other mouse strains or to animals with a longer duration or greater severity of diabetes. Additionally, oxidative stress and selenoproteins in glomerular cells other than podocytes may be important in the development and progression of nephropathy.

## Conclusion

The loss of podocyte selenoproteins does not enhance nephropathy or oxidative stress in C57BL/6 mice after 6 months of STZ-induced diabetes. Either podocyte selenoproteins are not important in protecting against diabetic nephropathy, or additional antioxidant proteins compensate for the absence of selenoproteins. As with any model system, the results may not be transferable to other diabetic models or to diabetic nephropathy in humans.

## Competing interests

The authors declare that they have no competing interests.

## Authors' contributions

MNB participated in the design and execution of all experiments and drafted the manuscript. JY participated in the mouse breeding, genotyping and immunohistochemistry. MAS participated in the analysis of kidney function and histology. KAB participated in mouse breeding, genotyping and induction of diabetes. MJB participated in conception of the study and helped draft the manuscript. BAC participated in conception of the study, generation and breeding of the floxed mice, and helped draft the manuscript. FCB participated in conception and design of the study, data analysis, and helped draft the manuscript. RJK participated in conception and design of the study, data analysis, and helped draft the manuscript. All authors read and approved the final manuscript.

## Pre-publication history

The pre-publication history for this paper can be accessed here:


